# The Contagion of Unethical Behavior and Social Learning: An Experimental Study

**DOI:** 10.3390/bs13020172

**Published:** 2023-02-14

**Authors:** Yefeng Chen, Yiwen Pan, Haohan Cui, Xiaolan Yang

**Affiliations:** 1 School of Economics, Zhejiang University, Hangzhou 310027, China; 2 Interdisciplinary Center for Social Sciences, Zhejiang University, Hangzhou 310027, China; 3 School of Business and Management, Shanghai International Studies University, Shanghai 201620, China; 4 Academy of Financial Research, Zhejiang University, Hangzhou 310027, China

**Keywords:** unethical behavior, social learning, self-serving bias, motivated reasoning, quantal response equilibrium

## Abstract

Unethical behavior is discovered that is more contagious than ethical behavior. This article attempts to propose one of the possible underlying mechanisms—people may have underconfidence bias in information updating due to motivated reasoning, and such bias exhibits in a different direction compared to the overconfident bias documented in the literature on ethical environment, which generate the asymmetric pattern in contagion. This study designs an experiment which relates the unethical behavior to social learning, where a series of subjects with private information about penalty decide sequentially whether to conduct unethical behavior publicly. This study adopts a quantal response equilibrium to construct a structural model for estimation of the bias. In total, 162 university students participated in our experiment and the results confirm the asymmetric patterns that people rely more on others’ precedent decisions rather than their private signal; therefore, the bias facilitates the contagion. This study also tests two punishment systems in the experiment and the results suggest a policy: slightly increasing penalties for the “followers” in the early stages would effectively suppress the contagion.

## 1. Introduction

A lot of empirical or experimental literature has found a fairly strong contagion effect in unethical behavior, with respect to dishonesty [1,2,3], corruption [4,5,6,7], tax evasion [8,9,10], doping [11], theft [12] and general crime [13]. In particular, it is worth noting the asymmetric patterns between ethical and unethical behaviors. Some literature finds that unethical behavior is more contagious than ethical behavior [14]. The literature, such as [1], typically describes cost-benefit, social norms, saliency, etc., as the mechanisms by which the unethical contagion occurs, but these mechanisms could also be applied to the contagion of ethical behavior, which might not directly address the asymmetries.

Why are unethical behaviors more contagious? What are the micro channels behind it? This article tries to propose one of the possible mechanisms which focuses on the asymmetries—people may have bias in information updating, especially when unethical behavior meets uncertain penalties. Additionally, the bias may exhibit underconfidence rather than the common overconfidence in an ethical environment, which generate the asymmetries. This study tries to test the hypothesis above.

Unethical behavior may cause potential financial or psychological penalties, which are the crucial distinction between unethical and ethical behaviors. Moreover, if the existence and severity of penalties involve uncertainty [15], predecessors’ behaviors could create an externality for followers’ belief processing—public history of behaviors could serve as signals about the actual state, such as whether the punishment occurs and how severe it is. After observing these signals, the decision-maker will have a greater incentive to follow the predecessors, at which point the contagion occurs.

This kind of logic has been widely discussed in the social learning literature [16,17] and has been applied in various contexts, such as investment, fashion, etc. (See [18] for a recent survey.) Other examples of the literature also document the relation between beliefs and contagion in the real world. Refs. [19,20,21,22] find that social influence or social information can strongly facilitate the unethical behaviors. Refs. [23,24] find that fake news typically spreads much faster than real news. A large-scale field experiment by [25] finds that belief bias is one source of the spread of discrimination.

The social learning experiments have also systematically found overconfidence bias in belief updating. Ref. [26] conducts a thorough meta study of 13 experiment papers on social learning, and finds that people rely more on their own private information and ignore the information from others’ decision. This kind of overconfidence bias facilitates the information aggregation and limits the contagion. However, this study suggests that the direction of bias is affected by the frame of ethicality. In unethical environments, people might be underconfident and rely more on others instead, which stimulates the contagion.

Why would the bias occur in the opposite direction under unethical behavior? Two possible psychological reason mentioned in the literature could explain the changes of the bias direction—motivated reasoning and bystander effect. Motivated reasoning suggests that people will be willing to put a great deal of effort into maintaining a positive self-image [27]; furthermore, they will try to find reasons or uncertainty in the environment to justify their self-interested behavior. These behaviors especially occur during the process of gathering or interpreting information [28], which is also referred to as motivated Bayesian. For example, people just need a little information to believe that their desired moral judgment is right [29,30], they will overestimate positive information while underestimating negative information [31], and they will often distort beliefs about a random outcome for self-interested purposes, such as believing that a statistically fair outcome is unlikely to occur [32]. Another psychological mechanism is known as the bystander effect. The psychological literature has found that moral responsibility is shared among groups and that this responsibility diminishes as the size of groups increases [33,34]. Refs. [35,36] finds that moral responsibility could be diffused when multiple parties are involved in an unethical decision and people believe that individual decisions would not change the overall consequences. Under these two psychological effects, people would biasedly trust the information sources that could help them shirk moral responsibility. In conclusion, unethicality triggers a conflict between self-interest and ethical norms. The incentive to preserve self-image prevents people from viewing uncertainty in a fair and rational way, resulting in people being more likely to follow the decisions of their predecessors.

In summary, the theory above answers the question of why unethical behavior contagion is more likely to happen, which also indicates the following hypotheses: first, predecessors’ behaviors can lead to contagion due the social learning, and therefore, an effective approach to inhibiting the contagion would be offsetting the influence of social learning at an early stage. Second, people will have a underconfidence bias in the learning process when facing an unethical decision, and such bias specifically facilitates the contagion of unethical behavior.

To test these hypotheses, this study designs an experiment that relates the unethical behavior to the social learning (a variant of the experiment in [37,38]). In the experiment, one of the participants has to take on a real effort-task and gain income from it. The other participants will decide sequentially whether to take away some part of his earnings. Stealing money has a probability of being punished, but whether the punishment occurs depends on the exogenous nature state. Participants cannot know the actual state when making decisions, but they would receive two kinds of information. One is an unbiased private signal about the state provided by the program, and the other is public history of predecessors’ actions. Participants need to infer the likelihood of punishment from these two sources of information and decide whether to steal or not. Our experiment design is almost exactly the same as the investment task in [38], with the only exception being the narrative frame and the source of subjects’ income. If agents are rational and self-interested, then they would not be influenced by the context and the experiment results should be identical to those in the literature.

To estimate bias in belief updating, this study adopts the structural estimation similar to [39,40]. The model uses the quantal response equilibrium to capture people’s errors in decision making [41] and tries to estimate how much the two types of information influence people’s decisions. To control for other-regarding preferences involved in the task, this study sets up a baseline treatment without any other public information, and extends [39] to allow estimation of other-regarding preferences. The experiment results find that people are biased in belief updating and confirms the theory discussed above. They believe that public information is more accurate and rely more on others’ precedent decisions in social learning. This result contracts the conclusion in the neutral behavior where people rely more on their private information [26]. 

In order to investigate what policy can curb the contagion of unethical behavior, this study sets up two different treatments with public information: constant punishment and rising punishment. According to the findings of simulations, rising punishment is effective in containing the spread of unethical behavior. Additionally, it only requires a minor increase at the start of the contagion to achieve good policy effects.

The paper is organized as follows: Section 2 describes the details of the experiment design and research hypotheses; Section 3 constructs the structural model to estimate the bias in social learning; Section 4 provides the experimental results and the simulation according to the structural model; and Section 5 concludes this article.

## 2. Experiment Design

### 2.1. Participants and Procedure

All the experiment sessions were conducted at the Basic Platform for Social Science Research at Zhejiang University. The computerized experiment was designed using the software program z-tree [42]. In total, six sessions, each including eight periods for all treatments, lasted approximately two hours. 

This study was approved by the Ethics Committee of Interdisciplinary Center for Social Sciences at Zhejiang University (approval number: 20170201). All subjects gave their informed consent for inclusion before they participated in the study. A total of 162 subjects participated and were given an average of RMB 65.1. In addition to the main session, 27 subjects participated in a robust treatment at a similar duration and with average payoffs the same as the main sessions.

### 2.2. The Experiment

The experiment tries to compose two elements—unethical behavior and contagion. Unethical behavior could have certain negative externalities for others, and with a certain probability, may face severe punishment ex post. Additionally, the contagion is reflected in the experiment as the classical social learning game [37,38]. Agents need to infer the probability of being punished from private signal and public history of others. They then make their own decisions, which might further influence the decisions of subsequent players. The setup and notation are as follows:

**Role A (“The Labor”)**—All subjects will be divided into two different roles, Role A and Role B. Role A is the “labor” and they will be asked to perform a series of real effort tasks. The real effort task in the experiment is a series of two-digit addition and subtraction calculations. Role A will generate 400 ECU after completing all the calculations.

**Role B (“The Taker”)**—Role B will get the initial payoff at the start of each period. In addition to an initial payoff, Role B also has the option of stealing part of Role A’s gains, which is the unethical behavior in the experiment (in the instructions, a neutral word “take” would be used instead of “steal”). There are several Role Bs in a group, and they will make their decisions one after another. The subsequent Role Bs can observe information about their predecessors’ decisions. In addition, the amount that each Role B steals will vary depending on the experiment treatment and the decision-making sequence. The leftover income after all steals is Role A’s final payoff.

**Uncertainty of Punishment**—There is a certain probability that stealing will be punished and those who commit stealing have to pay a fine. However, whether or not punishment occurs is the nature state, which is unobservable and the same for all Role Bs. Additionally, it will be determined by the computer before the period starts. The probability of punishment is 50% in each period and independent between periods. Subjects cannot accurately observe the exact state of nature when making decisions, but they can obtain private signals with information about the state of nature. After everyone in Role B have made their choices—and the state of nature is revealed—the punishment will be implemented according to the state of nature.

**Private Signal**—Although each subject does not exactly know the nature state, they all receive a private signal about the state. The private signals are independent of each other and cannot be observed by others. There are also two possibilities for private signals. Red signals indicate there will be a punishment for unethical behavior whereas green signals indicate no punishment. The private signals have 70% accuracy, which means that there is 70% chance that private signal is identical to the actual natural state.

**Public information**—Role B can also observe the history of all previous decisions, decision orders, the amounts of their stealing, and the possible fines to be paid. However, at this point, the nature state is not yet public.

**The Punishment Intensity**—Those who commit the stealing will be required to pay a fine in a punishment nature state. The amount of fine varies depending on the amount of stealing. This study defines the punishment intensity as the ratio of the fine to the amount of stealing. Two kinds of punishment intensity settings will be considered in the experiment: constant intensity and rising intensity. In the rising intensity, the ratio goes up when more players have chosen to steal in the history. In contrast, with constant intensity, the ratio is set to constantly to 2, and does not change with the decision history. The specific parameters used in the experiment are based on the rational equilibrium. See Section 2.3 for details.

**Figure 1:**
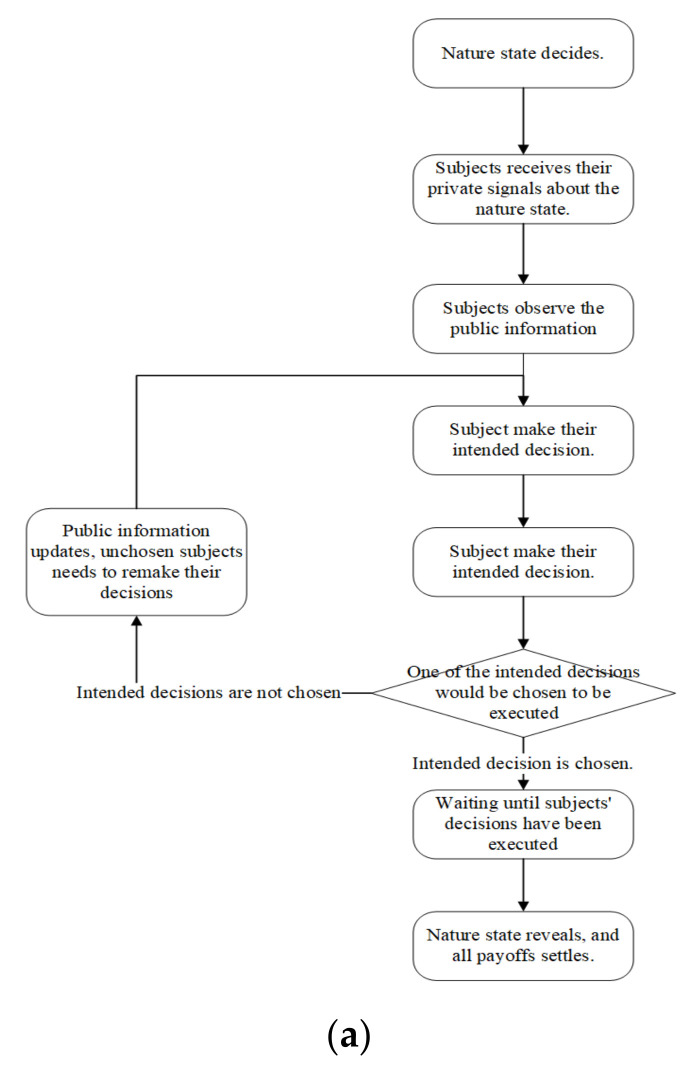

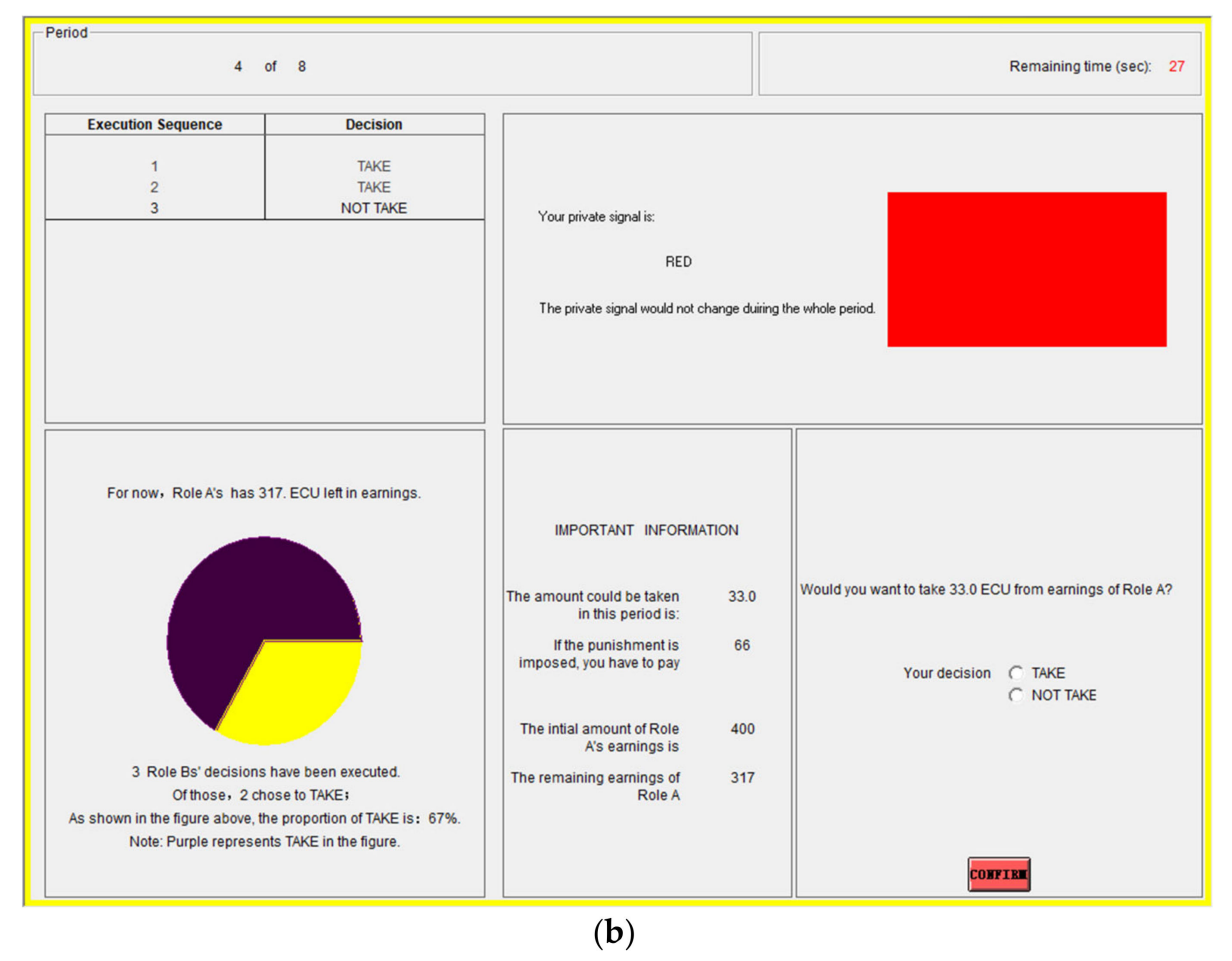
(**a**) Procedure Flow. (**b**) User Interface.

Every 9 participants would be allocated to a group. Eight of them are the Role B (“takers”) and the remaining one is the Role A (“labor”). Before the experiment starts, subjects are required to answer a series of test questions to ensure that they understand the rules. After completing the test questions, all 9 subjects have to take a real-effort task, which is identical to the task of Role A in the experiment. The task is to let all subjects experience the workload and tedium of task in the experiment. When the task ends, the experiment starts. A computer will randomly assign Role A or Role B to the subjects. The role will remain the same in the whole session. After the experiment, there is a questionnaire that collects demographical information related to the experiment. The procedure of the experiment is as follows (Figure 1 provides a flowchart of the procedure, as well as the user interface in experiment):

1. At the beginning of each period, a computer will determine the nature state based on a probability of 0.5. The results of the state are not revealed until all decisions for that period have been made;
2. The Role Bs receive their private signal for this period according to the state of nature. The private signals remain constant throughout the whole period. Private signals are displayed in the upper-right corner of the user interface;
3. Then Role Bs begin to make decisions. There are 8 Role Bs in a group, so the period is divided into 8 rounds for each decision made by a Role B, one after another. In the first round, subjects obtain their information in the user interface: private signal in the upper=right corner, the status of Role As remaining earnings in the lower-left corner, and the amount that can be stolen and possible fine in the lower-right corner. Once all 8 subjects have made their decisions, a computer will randomly select 1 of them to execute his or her decision. The subject whose decision is executed will receive a notification and will not participate in subsequent rounds in this period. The remaining unchosen decisions would not be executed in this round, but the subjects can re-make decisions in subsequent rounds;
4. In the second round, those remaining continue to make decisions. However, the stealing amount and the corresponding fine may change depending on the history of executed decisions. At the same time, public information is received in the upper-left corner of the Role B interface announcing the decisions made by the subject in the previous round. The new taking amount, the fine, and the public information are all the same for each Role B. Once all 7 Role Bs have made their decisions, a computer randomly selects 1 subject to execute his or her taking decision. The procedures are repeated in subsequent rounds until all subjects’ decisions have been executed in this period;
5. At the end of this period, the nature state will be revealed. The payoffs of all subjects including Role A will be settled.

During the whole session, Role A cannot participate in the decision-making described above. They are required to complete all the real effort tasks correctly and can know their remaining labor gains only after all decisions have been executed. In each round, the Role A needs to complete several 2-digit calculations and only after all the answers are correct could he or she move on to the next task. Role A also has to complete each calculation within 15 s, and failure to do so will result in a warning. Role A will complete hundreds of problems during an average two-hour session. The tasks of Role A are designed to be relatively tedious to reinforce the unethicality of Role Bs’ taking behavior. To further ensure the robustness of the task, this study also implemented a treatment that subjects are taking from a charity pool instead of labor income from a subject, and it did not change the main findings.

### 2.3. Treatments

To better control for other-regarding preferences involved in the task and identifying the bias in belief updating, this study sets up a baseline treatment with no public information. The other two treatments are provided with public information, but with different punishment intensity.

Table 1 shows the treatment settings of the experiment.

The experiment used a within-subject design in which all subjects were to participate in all three experimental treatments. To prevent learning effects in the experiment, the baseline Treatment 1 had to be conducted at the very beginning. The other two treatments were randomly ordered after Treatment 1. Therefore, half of the sessions were conducted in the order of Treatment 1 (baseline), Treatment 2 (constant intensity), and Treatment 3 (rising intensity); the other half of the sessions were in the order of Treatment 1, Treatment 3, and Treatment 2. In addition to these three treatments described above, this study also implemented a robust treatment replacing a charity pool with Role A.

### 2.4. Rational Equilibrium

This study introduced the classical results from the social learning literature for risk-neutral rational agents. The equilibrium results differed between constant punishment intensity and rising punishment intensity—where, in rising intensity, the degree of polarization is defined as the absolute difference between the number of subjects who choose to steal and the number of those who choose not to steal.

**Result 1.**  *Constant Punishment Intensity [16,17]: If the punishment intensity is fixed and the degree of polarization is greater than 2, Role B will ignore private signals and follow the majority of the predecessor.*

The specific proofs of Result 1 and Result 2 below are exactly the same with the literature cited in parentheses; they are consequently omitted here. The following is a simple example of Result 1: when there are only two historical decisions in public information, the first two agents both choose *a*, and the third agent’s private signal is *b.* The first two decided according to their private signal. By Bayesian rule, $P\left(A|a,a,b\right)=\frac{p^{2}\left(1-p\right)}{\left[p^{2}\left(1-p\right)+{\left(1-p\right)}^{2}p\right]}=p=0.7>0.5$, the third agent will choose to follow the public information, no matter what private signal the subject gets.

The intuition behind this result is as follow. The history of predecessors is a series of signals about the state of nature. The higher the proportion of taking behavior in the history, people are more likely in a state that punishment would not occur. Since the intensity is constant at 2, so if the belief is greater than a constant threshold the expected benefit of stealing is greater than the cost. For example, if one assumes that the belief that there is probability *p* of not being penalized, the expected benefit of taking 1 point is *p*, and the cost is $2\times \left(1-p\right)$. Therefore, when p>2/3, the cost is greater than the benefit. When the belief inferred from public information is stronger than the accuracy of private signal, then private signal would be ignored. No one in society will believe that their behavior will be punished, so contagion occurs. This leads to Hypothesis 1.

**Hypothesis 1.**  *With constant punishment intensity, if the degree of polarization is above a threshold (2), then unethical behaviors would be contagious.*

The sentencing rule in reality is similar to constant punishment intensity. The penalty is mainly determined by the damage they cause or the amount of stealing. However, according to Result 1, the rule is not effective for stopping the contagion. In contrast, in rising intensity, the fine changes and adjusts according to the history.

**Result 2.**  *Rising Punishment Intensity [43]: When the fine* $F_{t}=e/\mathrm{Pr}(\mathrm{punish}\;|\;\mathrm{history})$*, Role B will follow private signals and ignore the public information*.

The intuition behind this result is as follow. If the expected amount of fine $F_{t}$ is equal to the amount of stealing e, $E\left(F_{t}\right)=\mathrm{Pr}(\mathrm{punish}\;|\;\mathrm{history})\cdot F_{t}=e$, the value of public information would be offset by the increasing in fine completely, then contagion would not happen. This leads to our Hypothesis 2.

**Hypothesis 2.**  *In the case where punishment dynamically adjusts according to decision history and the fine increases gradually as polarization rises, people make decisions based only on their own private signals, and the contagion is restrained.*

The Result 2 suggests that, in order to prevent contagion, one must take the changes in public beliefs into accounts. And it should progressively increase the penalty accordingly. Two treatments are set up based on the hypothesis. The amounts of stealing and fines will vary with treatments. The amounts of stealing would decrease as the polarization increases, which are the same for all treatments. Such setting is to represent the possible scarcity and competition between potential takers. Under a constant intensity, the fine is set at two times the amounts of stealing. In the rising intensity, the amounts of stealing and the fines will be based on the relationship in Result 2, $F_{t}=e/\mathrm{Pr}(\mathrm{punish}\;|\;\mathrm{history})$. The details are shown in the Table 2.

### 2.5. Behavioral Factors: Preferences and Bias

The canonical equilibrium in social learning can also be applied to ethical or neutral behaviors. Therefore, the theory could not address the asymmetries between ethical and unethical behaviors. The assumptions of risk-neutral rational Bayesian disallow lots of behavioral channels induced by the unethicality, and so, it needs to incorporate serval behavioral patterns into models. First, the unethical behaviors would generate negative externalities to others, so the other-regarding preferences would involve in the decision-making. Second, the uncertainty of punishment also associates with the risk preferences. This leads us to the following research Hypothesis 3.

**Hypothesis 3.**  *Risk preferences and other-regrading preferences will play a larger role in unethical decision.*

The psychological literature suggests that when people are faced with unethical decision, they engage in motivated reasoning and behave as a motivated Bayesian during belief updating. Different sources of information do not have the same impact on beliefs. During the information processing, people have two different incentives. On one hand, they want to gain the profits from unethical behaviors. On the other hand, they want to avoid taking the responsibility for creating negative externalities or imposing harm on others, and they also want to lessen the regret when they are punished. When multiple sources of information are present, people are more inclined to believe in the source that takes less psychological responsibility. If there is a conflict between private signals and public information, people are more likely to trust others’ decisions than their own private information, which might give them the impression that they are not pivoting in making this unethical action. This is similar to the “bystander” effect mentioned earlier, which suggests moral responsibility is shared among the group. Thus, bias can arise during belief updating. People would not treat public and private information with the same weight. The public information has a greater impact on belief updating. Therefore, unethical behavior could be more contagious than other neutral behavior.

Since the bias in belief updating is difficult to measure directly in experiments, this study constructs a structural model to estimate the bias based on [39,40], as detailed in the next section. The model captures the bias in two ways: the base rate fallacy (BRF) and irrational expectations. The base rate fallacy is introduced by [44]. Agents with such fallacy would be more “stubborn” and give more weight to their own private signals while relatively ignoring public information. In the case of unethical behaviors, the bias would be in the opposite direction. The second approach is irrational expectations. Under rational expectations, the rationality of oneself is the same as his or her perceived rationality of others. However, people may have irrational expectations and systematically believe that others’ rationality are higher than their own. They expect themselves to be more likely to make mistakes than others.

**Hypothesis 4.**  *People will be biased in belief updating. When making unethical decisions, people would put more faith in others’ decision information than their own private signal.*

## 3. Structural Estimation

To validate the belief updating mechanism and to estimate subjects’ bias in belief updating, this study needed to construct a structural model. This section is organized as follows: Section 3.1 starts with preferences and incorporates risk preferences and other-regarding preferences; Section 3.2 focuses on the Role Bs’ decision process given private signal and public information; Section 3.3 elaborates on how public beliefs updates according to the new decision information and constructs a structural model; and Section 3.4 describes how to incorporate the bias in belief updating in the model.

### 3.1. Preferences

Risk preferences and other-regarding preferences are related to the decision in the unethical behaviors, so these two are both included in our preferences settings. In this paper, the constant relative risk aversion (CRRA) utility is chosen as the Bernoulli utility function,

(1) $$u\left(x\right)=\frac{x^{\rho }-1}{\rho }$$

where 1−ρ is relative coefficient of risk aversion. When ρ=1, the agent is risk-neutral; when ρ<1, the agent is risk averse, especially when it is negative. 

And the final utility depends on the Bernoulli utility gained from my payoff and other’s payoff, which captures the other-regarding preferences.

(2) $$V_{x}\left(x,y\right):={\bar{V}}_{x}\left(u\left(x\right),u\left(y\right)\right)$$

where x is Role B’s payoff, and y is Role A’s payoff. Additional settings are introduced in preferences to capture people’s judgments of fairness. The most commonly used setting for fairness is the inequity aversion proposed by Fehr and Schmidt (1999). Role B are more likely to commit unethical behavior if they perceive stealing as fair and acceptable because Role A might have more payoffs than him or her. The specific form is as follows.

(3) $$V_{x}\left(x,y\right)=u\left(x\right)+\mu_{1}\cdot \max\left\{u\left(x\right)-u\left(y\right),0\right\}+\mu_{2}\cdot \max\left\{u\left(y\right)-u\left(x\right),0\right\}$$

Two parameters $\mu_{1}$ and $\mu_{2}$ represent the two kinds of coefficients of inequity aversion. When they are negative, it means that the behavior is inequality averse. Moreover, $\mu_{1}$ represents the agent’s aversion to having a higher gain than others, and $\mu_{2}$ is an aversion to others having higher gains than me.

As Hypothesis 3 states, risk preference ρ and other-regarding preferences should significantly explain the behavior of the subjects. Specifically, people should show a relatively strong risk aversion and also care about the gains of others in society.

### 3.2. Mistakes during Decision

In order to capture the mistakes, errors, or bias during the decision process, the quantal response equilibrium is introduced into our model [41]. In contrast to the Nash equilibrium, quantal response model assumes that people do not always make their optimal decision. There is some probability that people make mistake. The main intuition behind quantal response equilibrium is that the error rate depends on the utility difference between the two decisions. People are less likely to choose a sub-optimal alternative when the utility difference is quite large. The larger the difference, the lower the probability of mistake. When utility gained from two options are closed, it might be hard or costly to distinguish these options. It is shown in the literature that quantal response equilibrium could more accurately describe and fit the behaviors in experiments [45]. The model here is based primarily on [39].

Before Role B decides, he or she observe the private signal and public information, and tries to make an inference about the natural state. He or she then estimates the expected utility gained from options and chooses the optimal one. In this subsection, the decision process is divided into two parts—the first derives the public beliefs from the public information, and then Bayesian updating belief by the private signal. The first part would leave the public belief undecided for the next subsection and this subsection would only focus on the second part. 

Suppose that there are two possible natural states w=A or w=B, and the corresponding best action for world A (world B) is α (β). And $p:=\mathrm{Pr}\left(s=A|w=A\right)=\mathrm{Pr}\left(s=B|w=B\right)$, which is the accuracy of private signal s. $p_{t}$ and the public belief that the natural state is A at period t. Given the public belief $p_{t}$ and private signal s, if the agent is a rational Bayesian, his or her belief about the natural state is

(4) $$\tilde{\mathrm{Pr}}\left(w=A|p_{t},s=A\right)=\frac{p_{t}p}{p_{t}p+\left(1-p_{t}\right)\left(1-p\right)}\;\tilde{\mathrm{Pr}}\left(w=A|p_{t},s=B\right)=\frac{p_{t}\left(1-p\right)}{p_{t}\left(1-p\right)+\left(1-p_{t}\right)p}$$

However, updating may be biased, so then the belief $\tilde{\mathrm{Pr}}\left(w|p_{t},s\right)$ might not follow the Bayesian rule. These cases would be discussed in Section 3.3. Therefore, the expected utility for action α or β is

(5) $$\begin{array}{l} \pi^{\alpha }\left(p_{t},s=A\right)\;:=E\left[V_{x}\left(\alpha |w\right)|p_{t},s=A\right]\\ \;\;\;\;\;\;\;\;\;\;\;\;\;\;\;\;\;\;\;\;\;\;\;\;\;\;\;\;\;\;=\tilde{\mathrm{Pr}}\left(w=A|p_{t},s=A\right)\cdot V_{x}\left(\alpha |A\right)+\tilde{\mathrm{Pr}}\left(w=B|p_{t},s=A\right)\cdot V_{x}\left(\alpha |B\right)\\ \pi^{\beta }\left(p_{t},s=A\right)\;:=E\left[V_{x}\left(\beta |w\right)|p_{t},s=A\right]\\ \;\;\;\;\;\;\;\;\;\;\;\;\;\;\;\;\;\;\;\;\;\;\;\;\;\;\;\;\;\;=\tilde{\mathrm{Pr}}\left(w=A|p_{t},s=A\right)\cdot V_{x}\left(\beta |A\right)+\tilde{\mathrm{Pr}}\left(w=B|p_{t},s=A\right)\cdot V_{x}\left(\beta |B\right) \end{array}$$

To simplify the notation here, $V_{x}\left(\alpha |A\right)\;:=V\left[u\left(x\left(\alpha |w=A\right)\right),u\left(y\left(\alpha \right)\right)\right]$ represents the utility when action α is selected, where $x\left(\alpha \right)$ and $y\left(\alpha \right)$ is the payoff for Role B and Role A.

Role Bs then make their decisions based on the quantal response equilibrium. The quantal response equilibrium is derived from the random utility model with an independent heterogeneous preference shocks ϵ, which is similar to Selten’s “trembling hands” [46]. Agent’s perceived utility ${\tilde{V}}_{x}$ is a weighted average between actual expected utility π and a logistic shock ϵ. The weights are determined by a coefficient λ which measures agent’s rationality. When λ tends to infinity, the agent is a rational Bayesian, and quantal response equilibrium degenerates to the Nash equilibrium. When Role B i chooses the action j, the perceived utility is

(6) $${\tilde{V}}_{it}^{j}\left(p_{t},s_{t}\right)=\lambda \pi_{i}^{j}\left(p_{t},s_{t}\right)+\epsilon_{it}^{j},\;\;j\in \left\{\alpha ,\;\beta \right\}$$

Then Role B i chooses the action α with the probability of

(7) $$\begin{array}{l} \sigma^{i}\left(p_{t},s\right)=\mathrm{Pr}({\tilde{V}}_{it}^{\alpha }>{\tilde{V}}_{it}^{\beta })=\mathrm{Pr}\left[\epsilon_{it}^{\alpha }-\epsilon_{it}^{\beta }>\lambda_{i}\left(\pi_{it}^{\beta }-\pi_{it}^{\alpha }\right)\right]\\ \;\;\;\;\;\;\;\;\;\;\;\;\;\;\;\;\;\;\;\;\;\;\;\;=\frac{1}{1+\exp\left[-\lambda_{i}\left(\pi_{it}^{\beta }\left(p_{t},s\right)-\pi_{it}^{\alpha }\left(p_{t},s\right)\right)\right]} \end{array}$$

### 3.3. Public Belief and Structural Estimation

The above model leaves the public belief $p_{t}$ undecided. Moreover, $p_{t+1}$ would be derived recursively based on the new information and public belief $p_{t}$ in ta. At the t period, Role B i made the action $h_{t}$ that is based on the public belief $p_{t}$. Before updating the $p_{t+1}$, it needs to derive the transition probability $T_{t}^{w=A}$ and $T_{t}^{w=B}$, where $T_{t}^{w=A}$ is the expected probability that action α is chosen give the natural state is w=A, and $T_{t}^{w=B}$ is the probability when w=B.

(8) $$\begin{array}{l} T_{t}^{w=A}=\mathrm{Pr}\left(h_{t}=\alpha |p_{t},w=A\right)\\ \;\;\;\;\;\;\;\;\;\;=\sigma \left(p_{t}.s=a\right)\cdot \mathrm{Pr}\left(s=a|w=A\right)+\bar{\sigma }\left(p_{t},s=b\right)\cdot \mathrm{Pr}\left(s=b|w=A\right)\\ \;\;\;\;\;\;\;\;\;\;=p\cdot \sigma \left(p_{t},a\right)+\left(1-p\right)\cdot \sigma \left(p_{t},b\right)\\ T_{t}^{w=B}=\mathrm{Pr}\left(h_{t}=\alpha |p_{t},w=B\right)=\left(1-p\right)\cdot \sigma \left(p_{t},a\right)+p\cdot \sigma \left(p_{t},b\right) \end{array}$$

The new public beliefs $p_{t+1}$ can be derived from $p_{t}$ and the transition probability $T_{t}$ through Bayesian updating.

(9) $$p_{t+1}\left(p_{t},h_{t}=\alpha \right)=\mathrm{Pr}\left(w=A|p_{t},h_{t}=\alpha \right)=\frac{p_{t}\cdot T_{t}^{w=A}}{p_{t}\cdot T_{t}^{w=A}+\left(1-p_{t}\right)\cdot T_{t}^{w=B}}\;\begin{array}{l} p_{t+1}\left(p_{t},h_{t}=\beta \right)=\mathrm{Pr}\left(w=A|p_{t},h_{t}=\beta \right)\\ \;\;\;\;\;\;\;\;\;\;\;\;\;\;\;\;\;\;\;\;\;\;\;\;\;\;\;\;=\frac{p_{t}\cdot \left(1-T_{t}^{w=A}\right)}{p_{t}\cdot \left(1-T_{t}^{w=A}\right)+\left(1-p_{t}\right)\cdot \left(1-T_{t}^{w=B}\right)} \end{array}$$

Based on this, given the agent’s preference parameters, it can obtain the public beliefs and the probability that each subject will take a specific action iteratively in each period. Accordingly, the structural model is then completely constructed and the required parameters are estimated using the method of maximum likelihood estimation.

(10) $$\max\log L=\sum_{t}I\left(h_{t}=\alpha \right)\cdot \log\sigma \left(p_{t},s_{t}\right)+I\left(h_{t}=\beta \right)\cdot \left(1-\log\sigma \left(p_{t},s_{t}\right)\right)$$

### 3.4. Bias in Belief Updating

Unethicality makes people act as motivated Bayesians treating information biasedly; people consequently form biased beliefs. To test this hypothesis, it needs to extend the structural model so that it can estimate the direction of the corresponding bias. Two ways would be used to model the bias in expectations. 

The first is the basic rate fallacy. The modeling here is similar to that of [39]. Bias is captured in probability $\tilde{\mathrm{Pr}}\left(w|p_{t},s\right)$, which is derived from a weighted Bayesian rule that places more or less weight on public beliefs than private signal. A new parameter τ>0 is introduced in the model indicating the degree of fallacy.

(11) $$\tilde{\mathrm{Pr}}\left(w=A|p_{t},s=a\right)=\frac{p^{\tau }p_{t}}{p^{\tau }p_{t}+{\left(1-p\right)}^{\tau }\left(1-p_{t}\right)}$$

When τ>1, agent assign more weight to the private signal than public beliefs; when τ<1, agent places more on public beliefs; and a rational Bayesian behaves as τ<1.

The second approach is irrational expectations, which is similar to [40]. Bias is captured in expectations about other people’s rationality λ, when agents incorporate the likelihood of other people making mistakes into their decisions. However, this expectation may be biased, when the perceived rationality might be more or less than the actual rationality. There are two λ in the model to differentiate their own rationality λ in decision-making from the perceived rationality $\bar{\lambda }$ about others in making an inference from public information. $\bar{\lambda }$ will affect the calculation of $\sigma \left(p_{t},s\right)$ used in public beliefs updating in subsection. When $\lambda =\bar{\lambda }$, people have rational expectations; when $\lambda >\bar{\lambda }$, people have overconfident expectations and believe that they make less mistakes than others; and when $\lambda <\bar{\lambda }$, people form underconfident expectations that others are more rational than themselves.

Based on the previous analysis, people may be more likely to trust others’ decision information in unethical behaviors in order to attribute moral responsibility to processors’ decisions. Accordingly, the coefficients in the structural model may also exhibit a pattern that τ is significantly less than 1 and $\lambda <\bar{\lambda }$.

## 4. Results

### 4.1. Contagion and Public Beliefs

This part first tests some results from classical social learning theory by examining how public beliefs affect the contagion of unethical behavior. According to Result 1, public information would trigger contagion when the degree of polarization is greater than or equal to 2. To test this hypothesis, this study selected the equilibrium-relevant subsample based on this condition. (The equilibrium test subsample is equivalent to the *relevant observations* in [38].) It is defined as a sample with a degree of polarization greater than or equal to 2 and the public information contradicts the private signal. The action would be counted as a contagion if subject violates his or her private signal and chooses to follow the dominated action in the public history. Table 3 shows the proportion of private signal violation in three treatments. Treatment 1 (baseline) shows the behaviors that were only affected by the risk or other-regarding preferences. It is also the control group for Treatment 2 and Treatment 3.

The results are generally consistent with Result 1 that public information leads to more contagion at constant punishment intensity. In Treatment 2 where public information is available, more people choose against their own private signals than people in Treatment 1. When stealing is the dominated action in public history, 64.14% of subjects comply with others and choose to steal. While in the baseline treatment, only 44.37% would choose to steal against the signal. The non-parametric test indicates a significantly higher proportion of private signal violations in Treatment 2 than in Treatment 1 (z = 7.937, *p* < 0.0001).

**Figure 2:**
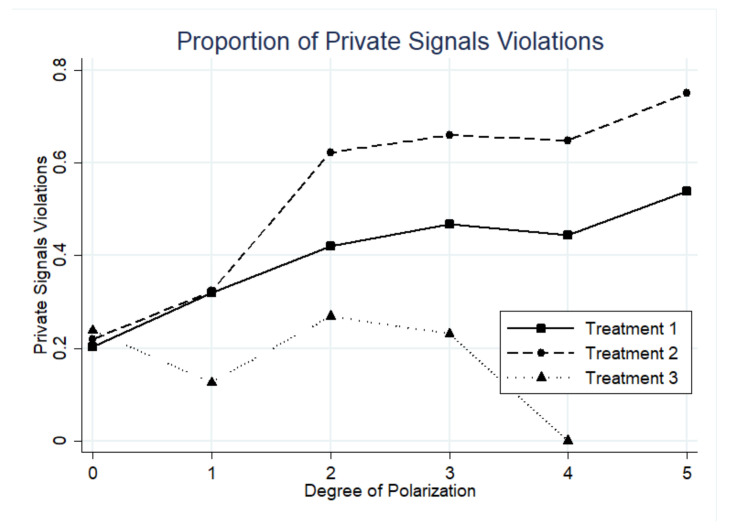
Proportion of private signals violations at each degree of polarization.

Another prediction from Result 1 is that contagion arises when the polarization is greater than or equal to 2. As is shown in Figure 2, when the polarization is less than or equal to 1, the proportion of private signal violations in Treatment 1 and Treatment 2 are very closed, but when the polarization exceeds 1, the gap between Treatment 1 and Treatment 2 increases significantly. In addition, as the degree of polarization rises, there is a tendency that the proportion of private signal violations increases and so do the stealing behaviors.

For Result 2, results are roughly in the same direction. When the punishment intensity rises according to the polarization, the proportion of private signal violations decreases from 64.14% in Treatment 2 to 26.04% in Treatment 3, even lower than in Treatment 1. The results of the non-parametric test indicate that the contagion is significantly inhibited by the rising punishment.

To further analyze the prediction of the theoretical model as well as to explore the relationship between subjects’ behavior and theoretically estimated public beliefs, a logit regression model is constructed as follows:

(12) $$Against_{it}=\Phi \left(\beta_{0}+\beta_{1}\cdot belief\left(H_{t},x_{it}\right)+\beta_{1}\cdot direction_{t}+\beta^{’}X_{it}+e_{it}\right)$$

where the $Against_{it}$ is a dummy variable for private signals violations, $belief:=\left|\mathrm{Pr}\left(w=B|H_{t},\;s\right)-0.5\right|$ is posterior probability of punishment based on the canonical social learning theory, and direction is dummy variable for the direction of the polarization.

The results of the regression in Table 4. The results show that in either Treatment 2 or Treatment 3, they could significantly predict subjects’ decisions, and as polarization increases, subjects are more likely to engage in contagion. In summary, it can conclude the following:

**Conclusion 1.**  *The experimental results are consistent with the predictions of Result 1. When public information is available, the contagion increases when polarization in public history rises.*

### 4.2. Rational Predictions and Actual Behavior

Result 2 based on theory is still quite different from the experimental results. Result 2 predicts that Treatment 3 and Treatment 1 should have similar proportions of private signal violates because the rising punishment exactly offsets the public information. However, the proportion of private signal violations in Treatment 3 is even lower than in Treatment 1 (no public information) and gets smaller as the polarization rises. When the polarization reaches 5, it drops to 0. This suggests that the punishment intensity is too severe if it is just based on the Nash equilibrium and Bayesian updating. It not only offsets the effect of public information, but also overly blocks other effects that are not related to the public information, such as risk or other-regarding preferences. Such severe punishment may not be feasible in reality since it requires strong law enforcement and it only allows extremely small tolerance for incorrect judgment.

Bounded rationality might be one of the possible explanations for the experimental results. According to the Bayesian rule, punishment intensity will grow exponentially. However, under bounded rationality intensity may not need to increase that quickly. The influences of risk and other-regarding preferences, as well as bounded rationality, will be discussed in the next subsections.

To further analyze the difference between actual behavior and Nash equilibrium, Table 5 summarizes the proportion of behaviors that match the Nash equilibrium in social learning. Since the worst prediction (completely random) is at 50% correct rate, this study sets 50% as a reference to calculate the relative match rate. 

Overall, the predictability of classic social learning theory is poor in unethical behavior. The lowest relative match rate is in Treatment 1 at 45.60%. However, when no public information is available, decisions are only affected by preferences and private signals, suggesting that the influence of risk and other-regarding preferences is very strong in unethical behavior. When a subject receives a green private signal, the match rate in Treatment 3 is 34.32%, which is even lower than that in Treatment 1. However, when the red signal is received, the match rate is at 70.66%. These two results indicate that, even when they receive green public information and the green private signal, some subjects still do not choose to steal. The other-regarding preferences might be the reason for this result. In addition, the highest match rate in Treatment 2 also confirms that Result 1 is more robust than Result 2. This leads us to the following conclusion:

**Conclusion 2.**  *There are still some disparities between the experimental results and Result 2. Risk preference, other-regarding preference, and bounded rationality—which are ignored in the classical model—might have significant influences on subjects’ unethical behavior.*

### 4.3. Behavioral Factors: Preferences and Bias

This subsection investigates the effects of serval behavioral factors, including risk preferences, other-regarding preferences, and bias in belief updating. The structural model in Section 3 would be used to estimate the results. Table 6 provides the major results:

The (1) shows the results of the preference-only model without the bias. The risk preference ρ are significantly smaller than 1 and negative, indicating that subjects exhibit strong risk aversion in their decision-making process. It confirms Conclusion 2 in the previous subsection. Subjects with high risk aversion are less likely to commit stealing or engage in unethical activity.

In terms of the other-regrading preferences, subjects exhibit partial inequality aversion. The disadvantageous inequality aversion $\mu_{2}$ is significantly smaller than 0, indicating that Role Bs shows inequality aversion when Role As have higher gains than him or her. However, the advantageous inequality aversion $\mu_{1}$ is positive, indicating that Role Bs might consider the equality is acceptable if Role As have lower gains than him or her. However, its effect size is considerably smaller than $\mu_{2}$. In addition, this study also conducts a survey on the subjects before the experiment, collecting subjects’ attitude toward the fairness of stealing behaviors. The results have great disparity to the actions in the experiments. The partial inequity aversion in the experiment might imply a self-serving justification of fairness, which suggest that stealing is perceived as fair and acceptable.

All these coefficients show that risk preferences and other-regarding preferences play an important role in people’s decisions, which confirms Hypothesis 3. In terms of bias in belief updating, the (2) and (3) columns in Table 6 provide the results in two different bias modeling settings—basic ratio fallacy and irrational expectation. The basic ratio fallacy measures agent’s stubbornness or how much weight subject relies on the private information in the updating. Rational Bayesian agents would be τ=1, but if τ<1, agents place more weights on the public information, and if τ>1 they are reluctant to change their mind and place more on the private signal. The results show that τ is significantly less than 1, indicating that subjects are “underconfident” about their own information. 

The irrational expectations measure the differences between agent’s rationality λ and his or her beliefs about others’ rationality $\bar{\lambda }$. If the agents have rational expectations about others, there should not be a systematic difference between these two. However, if λ is systematic greater than $\bar{\lambda }$, the agent would rely less on the public information and be “overconfident” in his or her decision. Otherwise, when $\lambda <\bar{\lambda }$, the agent would generally believe that public information is more accurate. The result shows that λ is significantly less than $\bar{\lambda }$, indicating that subjects generally think others behave more rationally than themselves, and thus are more likely to follow the public information.

These two results both confirm the biases in the same direction: people focus more on others’ information and relatively neglect their own private information. However, these results are inconsistent with the finding in ethicality-irrelevant social learning, such as [26,39]. Moreover, they find that τ is significantly larger than 1 and λ is systematic greater than $\bar{\lambda }$, showing a pattern of overconfidence. These inconsistencies suggest that the ethicality in social learning might change the direction of bias. This change of bias direction might imply a possible self-serving interpretation of information. When people choose to follow the precedents, they are less pivotal in the decision, and it is easier to justify their unethical behavior [36], which minimizes the guilt and regret they feel.

**Conclusion 3.**  *Subjects experience a biased interpretation of information when making a decision related to the ethicality. People rely more on public information and others’ decisions. People believe that others are more accurate, which enhances the spread of unethical behaviors. The direction of bias is not consistent with the bias in neutral behavior [26].*

### 4.4. Optimal Punishment Intensity

**Figure 3:**
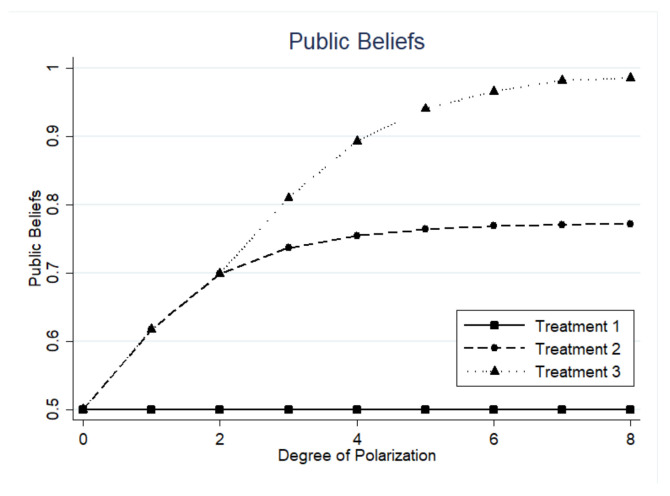
Changes in public beliefs at each degree of polarization.

Due to the neglect of several behavioral factors in the classic social learning model, Result 2 does not quite match the experimental results. After the structural model incorporates these factors, it tries to attain a better result in this subsection. In this instance, we plug the estimated coefficients into the basic model, which simulates the public beliefs at different degrees of polarization. As Figure 3 shows, public beliefs change faster and have a steeper slope when the degree of polarization is slightly larger than 2, but as the degree of polarization rises, the slope gradually flattens out.

In the next instance, it can take the estimated structural model as a benchmark and then find the optimal punishment intensity that exactly offsets public information. The simulated intensity could be constructed as follows:

(13) $$f_{t}=\arg\min{\left[\sigma \left(p_{t},e,f_{t}\right)-\sigma \left(0.5,e,2\right)\right]}^{2}$$

The first σ term in the optimization stands for the probability of stealing given public beliefs $p_{t}$, e is the stealing amount, and $f_{t}$ is the punishment intensity. The second term is the probability of stealing when there is no public information.

Table 7 gives the estimated optimal intensity at each degree of polarization. The optimal penalty intensity is much more moderate than the penalty intensity in the experiment. However, there is still a gradual upward trend in estimated intensity. On the other hand, it is also clear that public beliefs change quickest during the initial phase when polarization begins to rise. It is an effective timing to increase the intensity, which can also save the cost of policy implementation.

Thus, the experimental results are broadly consistent with Result 2—that raising the punishment could inhibit contagion of unethical behavior. However, because of the existence of bounded rationality and risk aversion, it is possible to have a more effective deterrence with only a minor increase at the beginning. It has some similarities to the nudge theory. Behavioral economics can sufficiently reduce the cost of policy implementation. Bias or irrationality can also serve as an effective policy tool.

**Conclusion 4.**  *The results are partially consistent with Result 2. Due to the existence of bounded rationality and risk aversion, it is more effective to slightly increase the punishment intensity at the beginning.*

## 5. Discussion

### 5.1. Conclusions

Contagion of unethical behavior tends to occur more rapidly than neutral behavior. This study focuses on the micro mechanisms behind these phenomena under the perspective of social learning. However, classical social learning theory, which based on risk-neutral rational Bayesians, cannot explain differences between unethical and neutral behavior. To better capture the characteristics of unethical behavior, this study tries to extend the social learning model to allow the estimate of several behavioral factors, such as risk preferences, altruism, and bias in information updating. These factors might lead to an increased propensity to follow the predecessors’ decisions and to commit unethical behavior. A quantal response equilibrium would be adopted to construct a structural estimation.

This study designs an experiment almost identical to the classical social learning experiment, with only a difference in the sources of subjects’ income. Subjects could increase their income at the expanse of reducing others’ earnings, which introduces the unethicality of their behaviors. Three treatments are set up varying the observability of others’ behavior and the punishment intensity. The results suggest some micro-channels underlying the contagion. People may misinterpret the accuracy of other people’s decisions and may perceive them to be more credible than themselves. Thus, they are more likely to blindly follow them. This direction of bias is the opposite to the bias in neutral task in the literature, which suggests that the direction of bias is strongly related to the context of the decision environment. Finally, the two punishment intensity treatments suggest that gradual adjustment of intensity is effective to control the contagion. Moreover, a slight increase in intensity in the early stages of contagion can be very essential.

### 5.2. Implications

The conclusions of this study have serval meaningful theoretical implication as following. Our findings suggest that different decision scenarios may influence the direction of bias in information updating. In ethically neutral scenarios, people will have overconfidence bias, whereas people will have underconfidence bias in unethical scenarios.

Additionally, our findings provide important insights into practices for controlling the transmission of unethical behavior. First, the micro channels suggest that contagion of unethical behaviors can occur more rapidly than neutral behavior. However, there is a constraint to how much people can distort their information and moral judgments. When there is less uncertainty in the environment, people are less likely to be biased. Therefore, a transparent and clear environment is needed to deter the contagion of unethical behavior. Second, gradually increasing the penalties for “followers” will be more effective than “heavily punishing the leaders”.

### 5.3. Limitations and Reconmendations

The conclusions of this study still have limitations. First, the internal validity of the findings relies strongly on the accuracy of the econometric models. This study did not directly observe or obtain the directions of bias, but instead adopts a structural model to identify it from subjects’ behavioral and questionnaire data. Further research can attempt to improve the experiment design to directly observe the direction of bias instead of relying heavily on complex statistical models. Second, the external validity of the findings is constrained by the student participants and abstract scenarios in the experiments, which makes it difficult to extend the findings to the real world. Further research may try to adopt a field experiment to obtain higher external validity.

## Figures and Tables

**Table 1:** Treatment Settings.

	Treatment 1	Treatment 2	Treatment 3
Public information	no	yes	yes
Punishment intensity	constant	constant	rising

**Table 2:** Punishment Intensity.

		Constant Punishment Intensity		Rising Punishment Intensity	
Polarization ^1^	Stealing	Fines	Intensity	Fines	Intensity
≤0	50	100	2	100	2
1	33	66	2	110	3.33
2	19	38	2	120	6.32
3	9	18	2	130	14.44
4	5	10	2	140	28
5	2	4	2	150	75
6	1	2	2	160	160
7	0.5	1	2	170	340

^1^ Polarization is the difference between the number of takers minus the number of non-takers in public information.

**Table 3:** Proportion of Private Signal Violations.

		Treatment 1	Treatment 2	Treatment 3
Proportion of private signal violations	Stealing Dominated in Public History (polarization ≥ 2)	44.37%	64.14%	26.04%
	Non-Stealing Dominated in Public History (polarization ≤ −2)	37.54%	65.17%	55.53%
Total	equilibrium-relevant subsample ^1^	642	788	593

^1^ The equilibrium test subsample is a sample with degree of polarization greater than or equal to 2 and the public information contradicts the private signal.

**Table 4:** Subjects’ Actions and Beliefs.

	Private Signal Violation	
	Treatment 2	Treatment 3
Public Belief	4.173 ***	4.974 ***
	(0.404)	(0.592)
Direction of polarization	0.248 ***	−0.126
	(0.0802)	(0.127)
Gender	0.147 **	0.0639
	(0.0706)	(0.0749)
Other control variables	√	√
Constant	−1.616 ***	0.549
	(0.470)	(0.544)

Standard errors are in parentheses. The control variables are mostly about the demographics, including age, ethnicity, Hukou, political party membership, and siblings. *** and ** denote significance at 1 and 5 percent levels, respectively.

**Table 5:** Relative Match Rate for the Nash equilibrium.

		Treatment 1	Treatment 2	Treatment 3
All observations	Red signal received	48.80%	74.22%	70.66%
	Green signal received	41.74%	60.96%	34.32%
	Total	45.60%	67.36%	52.70%

Relative match rate = (match rate − 50%) ÷ 50%.

**Table 6:** Structural Estimation Results.

		(1)	(2)	(3)	(4)
		Preferences Only	BRF	Irrational Expectations	Counting Strategy
Rationality	λ	6.3207	6.0747	6.9413	5.8401
		(1.4252)	(1.3585)	(1.4189)	(0.9683)
	$\bar{\lambda }$			8.4060	
				(1.9419)	
Risk and loss aversion	ρ	−0.0194	0.0132	−0.0946	−0.3678
		(0.1147)	(0.1136)	(0.1096)	(0.0830)
Inequality aversion	$\mu_{1}$	0.2291	0.2201	0.2918	1.4885
		(0.0921)	(0.0873)	(0.1051)	(0.4053)
	$\mu_{2}$	−0.5030	−0.4757	−0.6711	−2.4071
		(0.1595)	(0.1496)	(0.1996)	(0.7087)
Basic ratio fallacy	τ		0.9357		
			(0.0335)		
logL		−8394.0628	−8392.3498	−8390.8946	−8598.2991

Standard errors are in parentheses. This study also conducts a series of robustness tests of our structural model, including different utility function settings (exponential utility, prospect utility functions, and altruistic utility) and counting strategies (strategies that simply follow the majority in the historical public information), which are available to the reader upon request.

**Table 7:** Simulation of the Optimal Punishment Intensity.

		Estimated Optimal Intensity		Rising Intensity in Experiment	
Polarization ^1^	Stealing	Fines	Intensity	Fines	Intensity
0	50	100	2	100	2
1	33	111.01	3.36	110	3.33
2	19	86.30	4.54	120	6.32
3	9	59.04	6.56	130	14.44
4	5	34.02	6.80	140	28
5	2	20.48	10.24	150	75
6	1	8.65	8.65	160	160
7	0.5	4.42	8.85	170	340

^1^ Polarization is the difference between the number of takers and the number of non-takers in public information.

## Data Availability

Not applicable.
